# Outcomes after therapeutic SBE-ERCP for choledochojejunal/hepaticojejunal anastomotic stenosis after bile duct injury

**DOI:** 10.3389/fsurg.2025.1524479

**Published:** 2025-11-03

**Authors:** Yijun Shu, Pak-Lam Koo, Hao Weng, Li-Jia Pan, Ming-Zhe Weng, Ziyi Yang, Ming-Ning Zhao, Wen-Jie Zhang, Jun Gu, Wei Gong, Jia-Wei Mei, Yingbin Liu, Xue-Feng Wang

**Affiliations:** 1 Department of General Surgery, Xinhua Hospital, Affiliated with Shanghai Jiao Tong University School of Medicine, Shanghai, China; 2 Shanghai Key Laboratory of Biliary Tract Disease Research, Shanghai Jiao Tong University School of Medicine, Shanghai, China; 3 Shanghai Research Center of Biliary Tract Disease, Shanghai, China

**Keywords:** iatrogenic bile duct injury, single-balloon enteroscopy, ERCP, choledochojejunal anastomotic stenosis, balloon dilation

## Abstract

**Background:**

Choledochojejunal/hepaticojejunal anastomotic stenosis (CJS/HJS) is significant clinical problem associated with decreased survival postsurgery. Endoscopic retrograde cholangiopancreatography (ERCP) using single-balloon enteroscopy (SBE) is the first-line management strategy for such conditions. However, studies on the risk factors and outcomes of endoscopic management strategies for CJS/HJS in biliary duct injury (BDI) are extremely limited.

**Methods:**

We conducted a retrospective review of patients with symptomatic BDI who underwent choledochojejunal/hepaticojejunal Roux-en-Y anastomosis between April 2009 and April 2019. The primary endpoint was CJS/HJS recurrence. The secondary endpoint was early (i.e., emergent or unplanned) repeat SBE-ERCP (ER-SBE-ERCP). We also evaluated the details of initial therapy, complications, and treatment for CJS or HJS recurrence.

**Results:**

From April 2009 to April 2019, 112 patients were treated, and 45 (40.2%) BDI patients developed CJS/HJS. Operation type (*P* < 0.001), salvage surgery timing (*P* = 0.005), hepatic artery injury (*P* = 0.001), bile leakage after surgery (*P* < 0.001) and recurrent cholangitis (*P* < 0.001) were significantly associated with anastomotic stenosis. The overall CJS/HJS recurrence rate was 27.9% (12/43). Of all the patients, 79.1% (34/43) underwent balloon dilation at the anastomotic stenosis site; stent placement was performed in 33 of 43 patients (76.8%). The complication rate was 7% (3/43).Initial balloon dilation (*P* = 0.024) was associated with the proportion of patients requiring ER-SBE-ERCP. Predictors of CJS/HJS recurrence on bivariate analysis included initial balloon dilation (*P* < 0.001) and ER-SBE-ERCP (*P* < 0.001). On multivariate analysis, ER-SBE-ERCP was significantly associated with CJS/HJS recurrence, likely reflecting the presence of more severe lesions or higher baseline risks for recurrence, rather than being a direct cause of recurrence.

**Conclusions:**

Initial balloon dilation is associated with a decreased risk of CJS/HJS recurrence. ER-SBE-ERCP is more commonly performed in patients with severe anastomotic lesions or higher baseline risks for recurrence, which may contribute to the higher observed recurrence rates of CJS/HJS in this group.

## Introduction

Laparoscopic cholecystectomy (LC) is the standard treatment for symptomatic gallbladder disease and most of the around 700 thousand cholecystectomies done each year in China are laparoscopic. Biliary injury (1–3), bile leakage (4), biliary strictures, common bile duct (CBD) stone retention, postcholecystectomy syndrome and diarrhoea, vascular injury or haemorrhage, abscess formation, and bowel injury (5) are among the most usual complications of LC. Biliary injuries due to cholecystectomy affect long-term survival and quality of life negatively and create a considerable financial burden.

Cholecystectomy-related biliary injuries reduce long-term survival and quality of life and generate a significant financial burden. These patients frequently require complex management. Cutting a segment of the duct itself along with the bile duct disconnects the proximal biliary tree from the gastrointestinal tract and it needs surgical reconstruction to fix it. If an injury is noticed and fixed during LC, there are two choices: primary repair of the end-to-end duct or Roux-en-Y choledochojejunostomy.

Biliary strictures are a common complication following these surgeries. Choledochojejunal/hepaticojejunal anastomotic stenosis (CJS/HJS) is a significant late complication after postinjury salvage surgery that can cause hepatic failure, the need for transplantation and mortality. Historically, surgical reanastomosis has been the initial treatment option of choice for these patients, despite the fact that it is well known that reoperations in such circumstances are technically challenging and have a significant risk of complications (6, 7). On the other hand, the surgical method for bile duct anastomosis has been reported to be less effective and riskier than the percutaneous transhepatic therapy of CJS/HJS (8, 9). However, this treatment is invasive, and patients require long-term hospitalization (PTC for CJS/HJS require repeated sinus tract dilation/long-term carrying of external drainage). Alternatively, a minimally invasive endoscopic approach has been applied for the treatment of CJS/HJS. However, after digestive tract reconstruction, it is challenging to insert standard endoscopes into the far distal part of the small intestine and get access to the bile duct through the anastomosis. Single-balloon enteroscopy (SBE), recently developed, has made it possible for endoscopists to perform endoscopic retrograde cholangiopancreatography (ERCP) and several procedures connected to SBE-ERCP for CJS/HJS, such as serial stricture dilation with balloon or dilating catheters, followed by the placement of one or more side-by-side plastic stents (10, 11). Our centre has been researching the application of SBE-ERCP after complicated gastrointestinal tract reconstruction for a long time and is one of the single centres with the largest number of SBE-ERCP cases after gastrointestinal tract reconstruction in China (12–14); therefore, to determine the predictors of restenosis during the SBE-ERCP treatment period, we reviewed our experience with CJS/HJS over a 10-year period.

To the best of our knowledge, this is the largest study assessing the risk factors and outcomes of patients in whom clinical success was achieved using SBE-ERCP for CJS/HJS and evaluating the endoscopic variables as potential predictors of restenosis among patients undergoing SBE-ERCP.

## Materials and methods

### Study design and patient selection

With the approval of the institutional ethics committee, we conducted a retrospective cohort study of 112 adult patients (71 males; median age, 54 years; range, 24–67 years) who received treatment at Xinhua Hospital, Shanghai Jiao Tong University School of Medicine and its branch in Chongming from 2009 to 2019. Of these patients, 93 underwent laparoscopic cholecystectomy and 12 underwent open cholecystectomy. All patients were salvaged with Roux-en-Y choledochojejunostomy/hepaticojejunostomy. Among them, 45 patients who achieved technical and clinical success in treating CJS/HJS by SBE-ERCP and who were followed up for more than 24 months after the overall treatment were included in this study. The follow-up methods included blood or imaging tests or telephone interviews. Patients who initially received therapeutic procedures other than SBE, such as double-balloon enteroscopy, endoscopic ultrasound-guided or percutaneous procedures, were excluded.

### Selection criteria for SBE-ERCP

We diagnosed patients with CJS or HJS based on the clinical symptoms of cholangitis, MRI scans or cholestatic serum biochemistry assays showing dilated intra- or extrahepatic bile ducts that fulfilled the Tokyo guideline (15) and was confirmed by SBE. Contrast flow impairment or ductal narrowing without proximal biliary dilatation were not thought to be signs of CJS or HJS. Before treatment, all patients underwent abdominal computed tomography to exclude other diseases. Patients who underwent SBE-ERCP for additional evaluation had cholestatic serum biochemical tests, imaging studies, or both that revealed dilated intra- or extrahepatic bile ducts without any other clinical reason.

### Treatment protocol

We provided standard medical care to the patients after diagnosis, including antibiotic therapy and nutritional support. We obtained informed consent from all patients about the procedure and its possible benefits and risks. The patient was in the supine position during the procedure. We performed all procedures under either moderate sedation or general anaesthesia based on the general assessment results. Three endoscopists who had experience in performing more than 300 ERCP procedures used a side-viewing duodenoscope (TJF 160; Olympus Optical Co., Tokyo, Japan) for the procedure. We cannulated the choledochojejunal/hepaticojejunal anastomosis with a guidewire and injected contrast, which revealed CJS/HJS. We defined technical success of the initial treatment as balloon dilation with waist disappearance, stent insertion, or dilator placement at the CJS/HJS site with intended performance and complete removal of stones if present. We increased the balloon pressure until the waist of the anastomotic site resolved or to a maximum of 12 atm, depending on the balloon size suitable for the anastomotic bile duct diameter.

Then, stenting was carried out. We placed Amsterdam plastic biliary stents with a size from 7 to 10 Fr and length from 10 to 15 cm (Cook Medical, Bloomington, IN, USA) based on the cholangiography results. Patients with a clinically significant stricture underwent a gradual increase in the number of plastic stents (multistenting) placed side-by-side across the anastomotic site every 3 months for 1 year or for a shorter duration if the stricture resolved.

We defined clinical success as improvement of clinical symptoms or decrease of the serum transaminase/amylase level by more than 50% within 14 days after SBE-ERCP. We also considered balloon dilation with symptom improvement but without waist disappearance as technical and clinical success in this study.

Stent exchanges were typically done every 3 months, but if there were numerous stents in place, the interval was longer. Until improvements in cholangiography appearance, contrast flow, and serum biochemical tests were noticed, we continued complete endoscopic treatment for up to one year. Stricture resolution was defined as the absence of residual indentation at the anastomotic level at SBE-ERCP. The staff endoscopist decided the number of endoscopic treatments.

### Definition of recurrence

Patients usually require multiple endoscopic treatments to dilate the narrow bile duct to a diameter of×mm. We define CJS or HJS recurrence rates as follows: After the last SBE-ECRP treatment, we would follow up patients regularly (3, 6, 12, 24 months). During this period, if the patient developed anastomotic stenosis again at the choledochojejunostomy or hepaticojejunostomy site, which met the definition of anastomotic stenosis (including clinical symptoms, liver function tests, imaging studies), we considered it as a recurrence.

### Definition of early repeat SBE-ERCP (ER-SBE-ERCP)

When there was no other diagnosis, ER-SBE-ERCP was considered urgent, unscheduled, or early (happening before the expected or planned date for repeating it) if any one or more of these signs of blocked bile flow were present: blood bilirubin level more than twice as high as the normal maximum (or more than 1.5 times higher than before putting in a stent if it was already too high), fever over 38.0℃ with sudden belly pain, or imaging tests showing newly widened bile ducts.

### Outcome measures

The primary outcome of the study was CJS/HJS recurrence at the end of endoscopic therapy. Secondary outcomes were the time to CJS/HJS recurrence and how many patients had at least one ER-SBE-ERCP, both related to when they first had balloon dilation. The following variables were evaluated as potential predictors of CJS or HJS recurrence: sex, age, stricture type, liver function, time from operation to initial SBE-ERCP, balloon dilator size, presence of a waist at the anastomotic site and number of biliary stents inserted. We chose 3 months as the limit for how long it took to get the first SBE-ERCP because previous research used this time to split up the early time after surgery (16).

Tertiary outcomes were details on the initial therapy for CJS/HJS, complications of the initial therapy, and treatments for CJS/HJS recurrence. Complications related to the SBE-ERCP procedure and the severity of these complications were graded according to the American Society for Gastrointestinal Endoscopy (ASGE) guidelines (15).

### Statistical analysis

Continuous variables are presented as medians and ranges, and categorical variables are presented as frequencies and percentages. Significant differences were determined using Fisher's exact test. Associations between potential predictors and CJS/HJS recurrence were evaluated with bivariate and multivariate logistic regression analyses. The recurrence rates of CJS/HJS were contrasted using the log-rank test. A statistically significant difference was deemed to exist when the *p* value was less than 0.05. An odds ratio (OR) and 95% confidence interval (CI) are used to describe each result of the regression model. All statistical analyses were performed using SPSS.

## Results

### Patient and clinical characteristics

We identified 112 patients who underwent Roux-en-Y choledochojejunostomy/hepaticojejunal for bile duct injury (BDI) at different institutions. Table 1 shows the patient characteristics. The median age was 54 years (range, 16–92), and 63.4% of patients were male. The causes of BDI were LC injury in 93 (83%), open cholecystectomy injury in 12 (10.7%), and injury during other surgeries in 7 (6.3%) patients. The operation methods were choledochojejunostomy (*n* = 77) and hepaticojejunostomy (*n* = 35). The first presentation for patients with early stricture formation could be elevated liver biochemical markers (*n* = 39); other presenting symptoms could include abdominal pain (*n* = 30), jaundice (*n* = 35), and fever (*n* = 27). The median time from operation to initial SBE-ERCP was 102 days (range, 35–221). Twenty-two out of 77 patients (19.6%) developed CJS and 23 out of 35 (20.5%) developed HJS requiring therapeutic SBE-ERCP. Technical and clinical success were achieved in 43 (95.6%) patients after the initial treatment. Treatment failed in 2 patients due to afferent loop obstruction.

**Table 1:** Clinical characteristics of patients.

Clinical characteristics	*n* = 112
Age, years	54 ± 15
Male, *n* (%)	71 (63.4)
Aetiology, *n* (%)	
Laparoscopic cholecystectomy	93 (83.0)
Open cholecystectomy	12 (10.7)
Other operation	7 (6.3)
Operation, *n* (%)	
Choledochojejunostomy	77 (68.8)
Hepaticojejunostomy	35 (31.2)
Stricture type, *n* (%)	
CJS	22 (19.6)
HJS	23 (20.5)
No CJS or HJS	67 (59.8)
Clinical features, *n* (%)	
Abdominal pain	30 (66.7)
Jaundice	35 (77.8)
Fever	27 (60)
Liver function	
Abnormal	39 (34.8)
Normal	6 (5.4)
Time from operation to initial SBE-ERCP	
≤3 months	15 (11.6)
3–12 months	22 (19.6)
≥12 months	8 (7.1)
Imaging findings, *n* (%)	
BD dilation	33 (73.3)
BD stone	24 (43.4)

ERCP, endoscopic retrograde cholangiopancreatography; CJS/HJS, choledochojejunal/hepaticojejunal anastomotic stenosis; SBE, single-balloon enteroscopy; BD, bile duct.

### Classification of BDI

**Figure 1:**
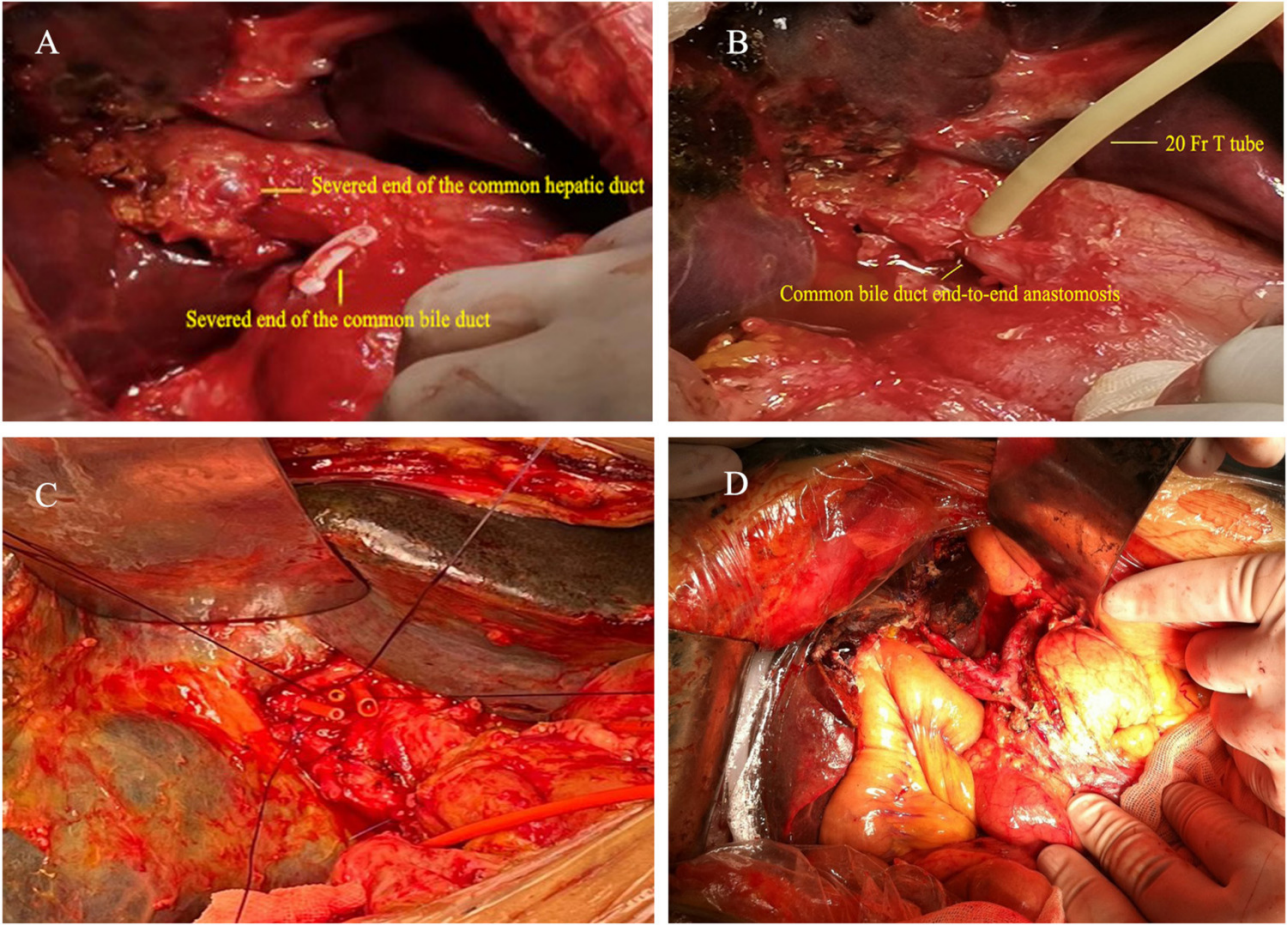
Surgical images of various biliary injuries. **(A**,**B)** In one patient, an intraoperative transection of the common bile duct was identified, and a hepatic duct-to-common bile duct anastomosis was performed with T-tube placement. **(C)** In another patient, end-to-end anastomosis of the common bile duct was performed. **(D)** Demonstration of hepaticojejunostomy (high biliary-enteric anastomosis).

The Strasberg classification (17) was used to categorize BDI after cholecystectomy. BDI was categorized as major (full or partial involvement of the CBD, common hepatic duct, or main segmental ducts at the porta hepatis) or minor depending on the degree of involvement (18). The BDI was Strasberg type B in 18 patients (16.1%), C in 19 patients (17%), D in 51 patients (45.5%), and E in 24 patients (21.4%). Partial injury or complete transection of the CBD was the most common injury site and the most common aetiology. The second most common site was the right hepatic duct (RHD) or right biliary system (38/112, 33.9%) (Figure 1).

### Details for the occurrence of CJS/HJS

Univariate analysis using the *χ*^2^ test and Fisher's exact test was performed to determine the relationship between CJS/HJS and perioperative variables. Several factors have been linked to an increased risk of CJS/HJS. CJS/HJS occurs due to blood supply disruption during dissection and recurrent bile leakage after salvage surgery. Operation type (*P* < 0.001), salvage surgery timing (*P* = 0.005), hepatic artery injury (*P* = 0.001), bile leakage after surgery (*P* < 0.001) and recurrent cholangitis (*P* < 0.001) were identified as the likely major factors associated with anastomotic stenosis (Supplementary Material Table 2).

### Details of initial therapy by SBE-ERCP

**Figure 2:**
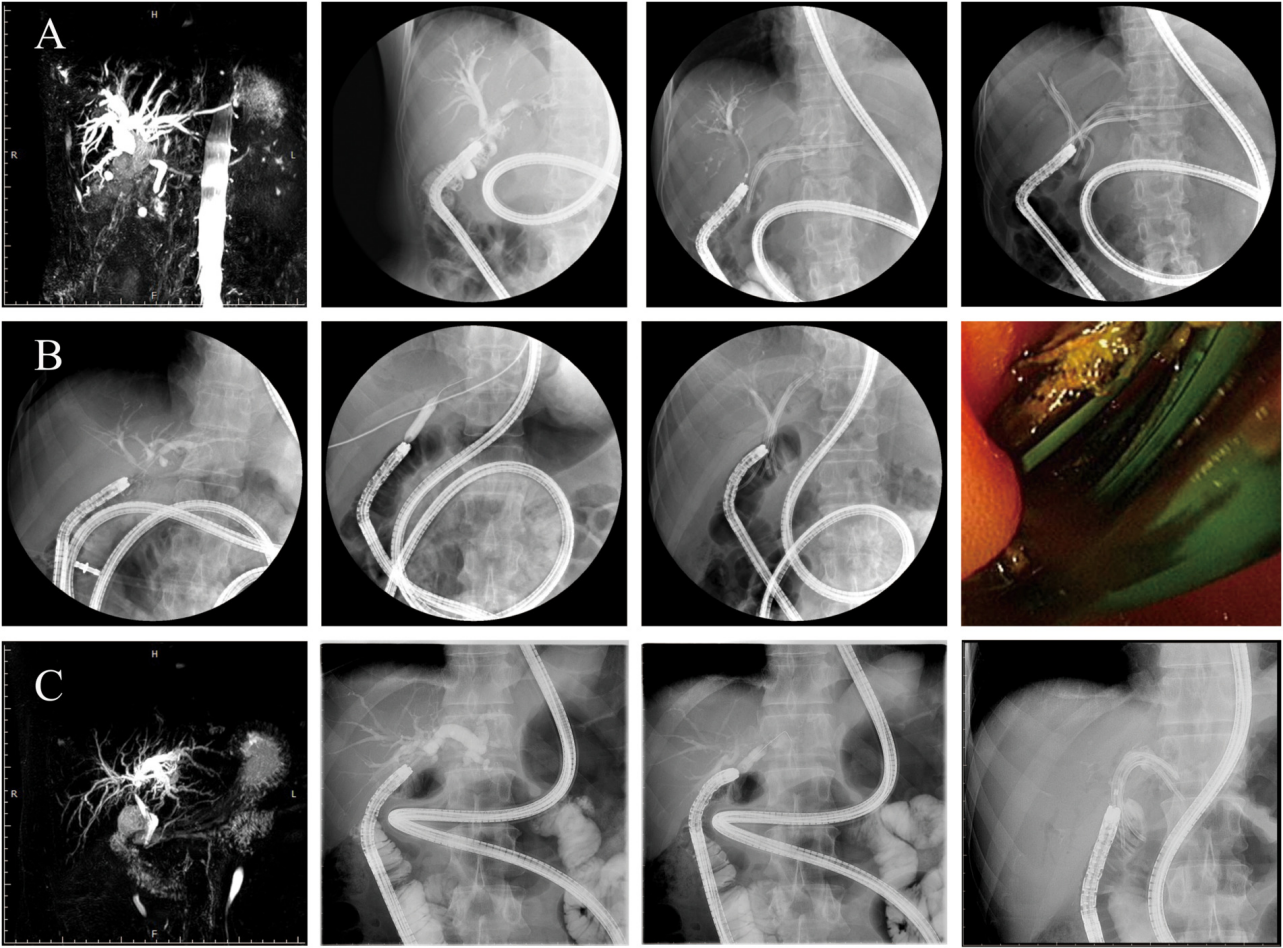
Operation procedure. The endoscopist collaborated with the nurse to inflate the scope and overtube as well as deflate and move the equipment, which were repeated to slowly advance the scope forward. Patients **(A–C)** represent three representative cases. In patient **(A)**, endoscopic cannulation revealed common bile duct stricture (CJS), and after balloon dilation, biliary stents were placed in both the left and right hepatic ducts. In patient **(B)**, no balloon dilation was performed; successful cannulation was followed by direct placement of a biliary stent. In patient **(C)**, cannulation revealed a stricture at the left hepatic duct anastomosis, and after balloon dilation, a stent was placed in the left hepatic duct. Biliary duct cannulation was completed, and cholangiography revealed CJS/HJS. The bilioenteric Roux-en-Y anastomotic stricture was treated by balloon dilatation.

For the original course of treatment, all CJS and HJS patients received single-balloon endoscopies (Figure 2). Table 2 shows the details of the SBE-ERCP procedure and findings. A balloon dilation catheter was used to treat 29 (64.4%) of the 43 patients, and a Soehendra dilation catheter was used to treat 5 (11.6%) of the patients. We used a 12-mm balloon dilation catheter in 10 patients (23.3%) of the 29 patients who received balloon dilation for anastomotic stenosis. We dilated 22 patients (51.2%) to a diameter <10 mm. Moreover, we dilated 24 patients (70%) until the waist of the anastomotic site resolved, and in 10 patients (29.4%), the anastomotic waist remained. We performed bile duct stone extraction in 24 patients. During a median follow-up of 1.4 years, we carried out a total of 135 ERCP procedures on the 45 patients who made up the research sample (excluding the final ERCP or upper endoscopy for stent removal). In our 83 ERCP procedures, we conducted endoscopic stenting, with 2 (1.5%) consisting of the placement of a single stent and 81 (60%) consisting of the placement of multiple stents (range, 2–4). We used stents with a diameter of 7–10 Fr and a length of 10–15 cm, with stents sized at 8.5 Fr × 10 cm being the most common. The complications included post-ERCP pancreatitis in 7 patients and postsphincterotomy bleeding in 2 patients. We managed these conditions conservatively. The median time from stent placement to removal was 105 days (range, 14–500).

**Table 2:** Details of ERCP findings.

Details of ERCP findings'	Patients (*n* = 45)	ERCP procedures (*n* = 135)
Successful CBD cannulation	43 (95.6)	125 (92.6)
Initial balloon dilation		
No	9 (20.9)	42 (33.6)
Performed or extended	34 (79.1)	83 (66.4)
Waist at the anastomotic site		
Resolved	24 (70.6)	56 (67.5)
Remained	10 (29.4)	27 (32.5)
Balloon dilator size		
4 mm	3 (8.8)	–
6 mm	7 (20.6)	
8 mm	10 (29.4)	
9 mm	2 (5.9)	
≥10 mm	12 (35.3)	
Diathermic dilation	9 (20.9)	42 (33.6)
Biliary stent placement		
1 stent	2 (4.7)	2 (1.5)
≥2 stents	31 (72.1)	81 (60)
None	10 (23.2)	42 (31.1)
Diameter, Fr, median (range)	7 (5–10)	–
Complications, *n* (%)		
Pancreatitis	2 (4.7)	
Post-ERCP bleeding	1 (2.3)	
Time from stent placement to removal, days, median (range)	105 (14–500)	

Outcomes		
Recurrence		
Yes	12 (27.9)	35 (25.9)
No	31 (72.1)	100 (74.1)
ER-SBE-ERCP		
Yes	15 (34.9)	40 (29.6)
No	28 (65.1)	–
Surgery required	6(14)	–

### CJS and HJS recurrence rates

**Figure 3:**
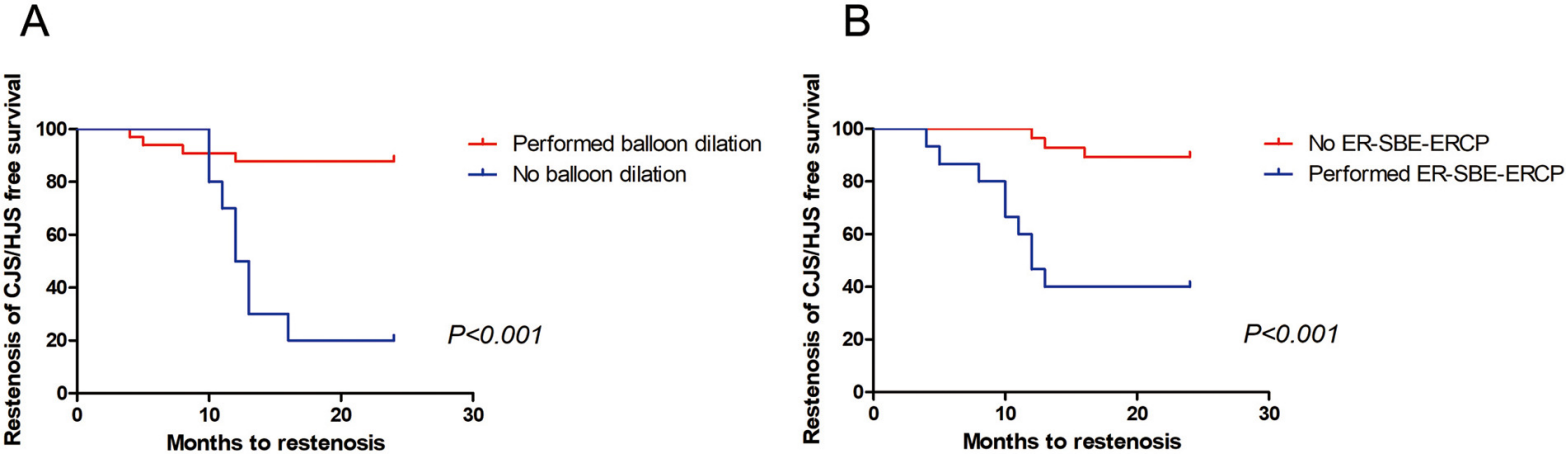
Time to CJS/HJS recurrence based on whether initial balloon dilation and ER-SBE-ERCP were performed. Kaplan–Meier curves demonstrated significant differences in recurrence-free survival, with patients who underwent initial balloon dilation (1A, *P* < 0.001) or at least 1 ER-SBE-ERCP procedure (1B, *P* < 0.001) demonstrating longer recurrence-free survival.

Table 2 also shows the primary and secondary outcomes. Following the original SBE-ERCP treatment for 43 patients who achieved clinical success, 12 patients (27.9%) experienced a recurrence of CJS or HJS. Four patients developed CJS recurrence, and 8 patients developed HJS recurrence, resulting in a 1 year recurrence rate of 100% (4/4) and 62.5% (5/8), respectively. The median time to CJS/HJS recurrence was 20.2 months (range, 4–24 months). Six patients with identified CJS/HJS recurrence underwent surgery for biliary anastomosis reconstruction. We performed univariate analysis to evaluate factors associated with CJS/HJS recurrence after the total treatment. Two variables were significantly associated with recurrence: initial balloon dilation at the anastomosis site and ER-SBE-ERCP (Table 3). In light of these results, we employed the Kaplan–Meier method to clarify the relationship between initial balloon dilation and time to recurrence. As shown in Figure 3, we discovered statistically significant variations in the time to CJS/HJS recurrence based on the timing of balloon dilation. Furthermore, we discovered that a greater percentage of patients who experienced recurrence needed at least one ER-SBE-ERCP (P0.001), indicating the potential significance of ER-SBE-ERCP for both short- and long-term patient outcomes. Regarding stent placement or size, there was no discernible variation in recurrence (*P* = 0.525).

**Table 3:** Univariate analysis of potential predictors associated with CJS/HJS recurrence.

Predictor variable	Recurrence (percentage of row)	c^2^	*P*
Sex			
Male	2 (16.7)	3.641	0.056
Female	10 (83.3)		
Age, years			
>60	7 (58.3)	0.142	0.707
<60	5 (41.7)		
Stricture type			
CJS	4 (33.3)	2.118	0.146
HJS	8 (66.7)		
Liver function			
Abnormal	11 (91.7)	1.707	0.191
Normal	1 (8.3)		
Time from operation to initial SBE-ERCP			
≤3 months	3 (25)	1.877	0.391
3–12 months	8 (66.7)		
≥12 months or never	1 (8.3)		
Initial balloon dilation			
Yes	**5** (**41.7)**	**14**.**071**	**<0**.**001**
No	**7** (**58.3)**		
Waist at the anastomotic site			
Resolved	2 (40)	2.642	0.104
Remained	3 (60)		
Stents placed			
Yes	10 (83.3)	0.405	0.525
No	2 (16.7)		
ER-SBE-ERCP			
Yes	**9** (**75)**	**11**.**793**	**0**.**001**
No	**3**(**25)**		

Bold values indicate statistically significant results (*p* < 0.05).

### ER-SBE-ERCP: associations and sequelae

Fifteen patients (34.9%) fulfilled the prerequisites for the ER-SBE-ERCP secondary endpoint. Notably, the stricture resolution rate among the 9 patients who needed at least one ER-ERCP was significantly lower than that of the 3 patients who did not require ER-ERCP after endoscopic therapy (*P* = 0.001). On bivariate analysis, the initial balloon dilation was the only factor that was statistically related to ER-SBE-ERCP (Supplementary Material Table 3). As a result, balloon dilation appeared to be significantly associated with a reduced likelihood of ER-SBE-ERCP procedures occurring sooner and more frequently. Furthermore, as previously stated, ER-SBE-ERCP was associated with a significantly higher CJS/HJS recurrence incidence.

### Multivariate modelling to identify predictors of CJS/HJS recurrence

To assess numerous potential predictors of CJS/HJS recurrence at once, we built multivariate logistic regression models. CJS/HJS recurrence was found to be considerably correlated with ER-SBE-ERCP (Table 4). ER-SBE-ERCP was significantly associated with CJS/HJS recurrence, likely reflecting the presence of more severe lesions or higher baseline risks for recurrence, rather than being a direct cause of recurrence.

**Table 4:** Univariate and multivariate analyses of clinical variables contributing to CJS/HJS recurrence.

Variable	Univariate analysis		Multivariate analysis	
	HR (95%CI)	*p* value	HR (95%CI)	*p* value
Sex (male vs. female)	0.703 (0.210–2.356)	0.568	–	
Age (<60 vs. >60 years)	0.318 (0.100–1.009)	0.052	–	
Stricture type (CJS vs. HJS)	0.458 (0.145–1.440)	0.181	–	
Liver function (abnormal vs. normal)	3.149 (0.491–20.20)	0.226	–	
Time from operation to initial SBE-ERCP (<3 months vs. >3 months)	7.139 (1.914–26.632)	0.122		
Initial balloon dilation (yes vs. no)	***0.047*** *(****0.011–0.199)***	***<0***.***001***	–	0.082
Waist at the anastomotic site (resolved vs. remained)	0.162 (0.022–1.214)	0.063	–	
Stents placed (yes vs. no)	1.288 (0.266–6.225)	0.731	–	
ER-SBE-ERCP (yes vs. no)	***0.077*** (***0.021–0.282)***	***<0***.***001***	8.565 (2.299–31.911)	0.001

Bold values indicate statistically significant results (*p* < 0.05).

## Discussion

The inability to prevent damaging the biliary tract and its blood supply during dissection causes biliary injuries linked to cholecystectomy. Acute cholecystitis, choledocholithiasis, anatomic variations in the biliary tree anatomy, urgent surgery, and inability to accurately identify the cystic duct prior to clipping or division are some of the factors that have been linked to an increased risk of CBD injury following cholecystectomy (3, 19, 20).

The most common site of injury in BDI patients is the CBD, followed by the RHD. Management of cholecystectomy-related CBD injuries may involve percutaneous, endoscopic or surgical interventions depending on the type of injury. Surgical interventions may be used for large lateral defects in major ducts, strictures refractory to other treatments and nearly any complete transection or ligation. Roux-en-Y choledochojejunostomy is often the best treatment option for major BDI and provides excellent long-term outcomes. CJS/HJS is a significant late complication after Roux-en-Y choledochojejunostomy/hepaticojejunostomy with an incidence of 0.5%. Elevated liver biochemical markers may be the first presentation for patients with ligation or early stricture formation. Other symptoms may include abdominal pain (66.7%), jaundice (77.8%), and fever (60%). However, the accuracy of noninvasive imaging such as T-tube and magnetic resonance cholangiography in confirming CJS/HJS has been little studied and findings are controversial (18–20). Due to the clinical importance of CJS/HJS, its diagnosis in patients with abnormal liver function test results, especially when there is a radiological suspicion of CJS/HJS, is pivotal for effective treatment.

Due to the absence of the need for external drain placement in comparison to surgical or percutaneous modalities, SBE-ERCP is regarded as the first-line management choice for the majority of CJS/HJS patients (21, 22). In patients with complex postsurgical anatomy, SBE for ERCP is a safe and effective technique for biliary endotherapy. PTC was selected for patients who failed SBE-ERCP or could not tolerate general anesthesia.

The total ERCP success rate for biliary disease has been found to be 50%–94% with regard to the short-term outcomes of SBE-ERCP in patients with altered gastrointestinal anatomy (23–25). In contrast, the reported success rate of percutaneous transhepatic treatment and surgical reanastomosis for the treatment of postoperative bile duct stenosis, including CJS, has been reported to be 84%–99% and 83%–91%, respectively (26, 27). These results indicate that SBE-ERCP has a comparable success rate to percutaneous transhepatic treatment or surgery and its utility for cholecystectomy-related CBD injury has been increasingly reported.

In treating CJS/HJS patients, identifying the anastomotic site and cannulating the biliary duct can be difficult due to sharp angulation and lack of a cannula deflector. The technical and clinical success rate of SBE-ERCP for CJS and HJS in this study was 95.6%, which is acceptable compared to past reports.

Percutaneous transhepatic therapy for CJS or HJS and surgical reanastomosis have shown long-term outcomes with posttreatment restenosis rates of 11%–34% and 12%–23% (28, 29), respectively. The long-term effects of SBE-ERCP for CJS or HJS, however, have received relatively little attention. According to Sakakihara et al., there was a 21.9% (7/32) restenosis incidence following balloon dilation for CJS, whether a biliary stent was deployed or not. The average observation period following stent removal was 604.0 ± 368.6 days (30). In this study, the recurrence rate of CJS/HJS after total endoscopic treatment was 26.7% (12/45). Due to the different research designs and follow-up times, it is challenging to compare these studies, but the long-term results of SBE-ERCP treatment in this study and earlier reports were comparable to those of percutaneous transhepatic therapy or surgery. In contrast to surgical treatment, SBE-ERCP had a higher restenosis rate in this research. This might be because the research was small and there is still some uncertainty regarding the long-term effects of SBE-ERCP for CJS.

Recently, a number of therapeutic procedures employing SBE have been carried out for patients with CJS or HJS, including stent implantation, needle-knife precutting, and diathermic or nondiathermic dilation. In this study, 12 patients (35.3%) received balloon dilation to a diameter of less than 10 mm, which was performed on 79.1% of the patients with anastomotic stenosis. Patients with an intact papilla of Vater but changed gastrointestinal anatomy have been shown to be feasible, effective, and safe when undergoing endoscopic papillary large-balloon dilation using a balloon catheter smaller than 10 mm (31, 32). According to our statistical study, initial balloon dilation was associated with the recurrence of CJS/HJS, a longer time to recurrence, and fewer subsequent ER-SBE-ERCP procedures, suggesting that patients with more severe conditions might need more frequent interventions. A balloon width of 10 mm and 10 mm had no discernible impact on the anastomotic waist disappearance following balloon dilation. The patient's bile duct diameter at the moment of balloon dilation of the anastomotic site served as the basis for our choice of balloon diameter. These findings imply that, independent of balloon diameter, anastomotic stenosis may readily recur in patients with severe CJS and a residual waist at the anastomotic site even after balloon dilation. Patients who required at least one ER-SBE-ERCP procedure had a higher recurrence rate of CJS/HJS, which may indicate that those with more severe or recurrent cases are more likely to require multiple interventions. Regarding the reason annular anastomotic stenosis during ER-SBE-ERCP may increase vulnerability to trauma from the quick increase in the bile duct diameter during balloon dilation, it may be related to the possibility that ER-SBE-ERCP increases the chance of CJS/HJS recurrence. Such injuries may result in ductal oedema, which impairs bile flow around biliary stents, as well as modifications to the function and make-up of the biliary epithelium, which impairs flow through stents. Compared to single-balloon dilation, stent placement for CJS or HJS has a longer-lasting expansion impact. To avoid mechanical stimulation-induced occlusion, calculus formation, and granulation, the inserted stents must be withdrawn within a predetermined time frame (33). Regarding the quantity, size, and frequency of stent exchanges, a number of strategies have been outlined, but no ideal strategy has been found yet (34–36). The decision to stop using plastic stents is usually made when the stricture completely vanishes on cholangiography. Plastic stents are changed occasionally every 3–6 months. After extensive follow-up, new research by Costamagna et al. (37) showed the security and effectiveness of endoscopic therapy using multiple plastic biliary stent placement procedures for CJS. The rate of CJS/HJS resolution was 90.7% (39/43) and 2 cases of failed SBE-ERCP were excluded. The interval between the biliary stent's insertion and removal and the likelihood of CJS/HJS return was not significantly correlated. However, there were no limitations on the types of treatment and management techniques used in this retrospective research, such as the choice between scheduled and on-demand stent removal or balloon dilation and stent insertion. Clarification of the long-term effects of stenting for CJS and HJS requires additional analysis in comprehensive research.

Similar to earlier studies on SBE-ERCP, 3 out of the 43 patients (or 7%) who received the initial treatment for CJS and HJS experienced treatment-related complications. In this research, no other severe complications were found. One patient experienced delayed bleeding 2 days after the original SBE-ERCP with needle-knife precutting for CJS and endoscopic haemostasis by argon plasma coagulation using SBE was completed successfully. Several studies from high-volume centers have documented choledochojejunal anastomotic bleeding and perforation following diathermic dilation or stricturoplasty using a needle-knife (38–40). Although there was a tendency toward nondiathermic dilation in the current research, it is still unclear whether the diathermic approach for CJS is safe and acceptable.

There are a few restrictions on this research. The first goal was to assess only the long-term effects of SBE-ERCP for CJS/HJS. After receiving initial SBE-ERCP therapy, only patients who experienced technical and clinical success and underwent a minimum of a 6-month follow-up were included. Some patients who had been included in other studies were excluded from this research, and details of unsuccessful cases or patients with less than 6 months of follow-up were not evaluated. Therefore, it was not possible to fully avoid patient selection bias. Second, because this was a retrospective research, we were unable to determine the appropriate sample size or conduct a statistical power analysis.

## Conclusions

In summary, this research demonstrates the long-term effects of SBE-ERCP for CJS or HJS. The long-term effects of SBE-ERCP are comparable to those of surgery reanastomosis or percutaneous transhepatic therapy. Initial biliary balloon dilation appears to be associated with a decreased risk of CJS/HJS recurrence and The higher CJS/HJS recurrence rate in patients who require multiple ER-SBE-ERCP procedures is likely due to these patients having more severe anastomotic lesions or higher baseline risks for recurrence, rather than the procedure itself being a direct cause of recurrence.

## Data Availability

The original contributions presented in the study are included in the article/Supplementary Material, further inquiries can be directed to the corresponding authors.
