# Supplementary material for: Outcomes after therapeutic SBE-ERCP for choledochojejunal/hepaticojejunal anastomotic stenosis after bile duct injury
Source: Front Surg. 2025 Nov 3;12:1524479. doi: 10.3389/fsurg.2025.1524479 (PMC12620351; doi:10.3389/fsurg.2025.1524479)
Supplement: Supplementary file 1 [file Table1.docx]

| Supplementary Table 1. Strasberg-Bismuth Injury Classification of 112 patients | |
| --- | --- |
|  |  |
| Strasberg Classification | n (%) |
|  |  |
| A (cystic duct stump) | 0 |
| B | 18 |
| C (right aberrant duct injury) | 19 |
| D |  |
| RHD | 19 |
| LHD | 6 |
| CBD | 26 |
| E (complete transection of CBD) | 24 |
| Major bile duct injury | 18 |
| Minor bile duct injury | 6 |
| CBD, common bile duct; CHD, common hepatic duct; LHD, left hepatic duct; RHD, right hepatic duct | |
|  |  |
