# Supplementary material for: Outcomes after therapeutic SBE-ERCP for choledochojejunal/hepaticojejunal anastomotic stenosis after bile duct injury
Source: Front Surg. 2025 Nov 3;12:1524479. doi: 10.3389/fsurg.2025.1524479 (PMC12620351; doi:10.3389/fsurg.2025.1524479)
Supplement: Supplementary file 2 [file Table2.docx]

| Supplementary Table 2. Univariate analysis of potential predictors associated with CJS/HJS occurrence | | | | | |
| --- | --- | --- | --- | --- | --- |
|  |  | Stricture | | χ^2^ | *P* |
|  |  | Yes (n=45) | None (n=67) |  |  |
| Aetiologies | Laparoscopic cholecystectomy | 35(77.8) | 58(86.6) | 1.476 | 0.224 |
|  | Other operation | 10(22.2) | 9(13.4) |  |  |
| Operation | Choledochojejunostomy | 16(35.6) | 61(91.0) | 38.58 | <0.001 |
|  | Hepaticojejunostomy | 29(64.4) | 6(9.0) |  |  |
| Timing of Surgery | When damaged | 15(33.3) | 43(64.2) | 10.274 | 0.005 |
|  | < 1 week | 13(28.9) | 10(14.9) |  |  |
|  | > 1 week | 17(37.8) | 14(20.9) |  |  |
| With hepatic artery injury | Yes | 25(55.6) | 17(25.4) | 10.463 | 0.001 |
|  | No | 20(44.4) | 50(74.6) |  |  |
| Bile leakage after surgery | Yes | 20(44.4) | 7(10.4) | 17.006 | <0.001 |
|  | No | 25(55.6) | 60(89.6) |  |  |
| Recurrent cholangitis | Yes | 35(77.8) | 9(13.4) | 46.728 | <0.001 |
