# Supplementary material for: Outcomes after therapeutic SBE-ERCP for choledochojejunal/hepaticojejunal anastomotic stenosis after bile duct injury
Source: Front Surg. 2025 Nov 3;12:1524479. doi: 10.3389/fsurg.2025.1524479 (PMC12620351; doi:10.3389/fsurg.2025.1524479)
Supplement: Supplementary file 3 [file Table3.docx]

| Supplementary Table 3. Univariate analysis of potential predictors associated with ER-SBE-ERCP for CJS/HJS | | | |  |
| --- | --- | --- | --- | --- |
| Predictor variable | repeater-SBE-ERCP (percentage of row) | χ^2^ | *P* |  |
|  |  |  |  |  |
| **Sex** |  |  |  |  |
| Male | 8(53.3) | 0.882 | 0.348 |  |
| Female | 7(46.7) |  |  |  |
| **Age, years** |  |  |  |  |
| >60 | 6(40) | 0.002 | 0.964 |  |
| <60 | 9(60) |  |  |  |
| **Stricture type** |  |  |  |  |
| CJS | 7(46.7) | 0.186 | 0.666 |  |
| HJS | 8(53.3) |  |  |  |
| **Liver function** |  |  |  |  |
| Abnormal | 13(86.7) | 0.444 | 0.505 |  |
| Normal | 2(13.3) |  |  |  |
| **Time from operation to initial SBE-ERCP** |  |  |  |  |
| ≤ 3 months | 4(26.7) | 0.993 | 0.609 |  |
| 3 to 12 months | 7(46.7) |  |  |  |
| ≥ 12 months or never | 4(26.7) |  |  |  |
| **Balloon dilation** |  |  |  |  |
| **Yes** | **6(40)** | **5.062** | **0.024** |  |
| **No** | **9(60)** |  |  |  |
| **Waist at the anastomotic site** |  |  |  |  |
| Resolved | 6(66.7) | 0.091 | 0.763 |  |
| Remained | 3(33.3) |  |  |  |
| **Stents placed** |  |  |  |  |
| Yes | 12(80) | 2.088 | 0.148 |  |
| No | 3(20) |  |  |  |
